# Laboratory Response to Anthrax Bioterrorism, New York City, 2001

**DOI:** 10.3201/eid0810.020376

**Published:** 2002-10

**Authors:** Michael B. Heller, Michel L. Bunning, Martin E.B. France, Debra M. Niemeyer, Leonard Peruski, Tim Naimi, Phillip M. Talboy, Patrick H. Murray, Harald W. Pietz, John Kornblum, William Oleszko, Sara T. Beatrice, Joint Microbiological Rapid Response Team, New York City Anthrax Investigation Working Group

**Affiliations:** * New York City Department of Health, New York, New York, USA; † Centers for Disease Control and Prevention, Atlanta, Georgia, USA; ‡ Warfighting Concepts and Architecture Integration Division (J-8), The Joint Staff, Washington, D.C., USA; § Joint Program Office for Biological Defense, Falls Church, Virginia, USA; ¶ Naval Medical Research Center, Silver Spring, Maryland, USA; # Seymour Johnson Air Force Base, Goldsboro, North Carolina, USA

**Keywords:** anthrax, *Bacillus anthracis*, bioterrorism

## Abstract

In October 2001, the greater New York City Metropolitan Area was the scene of a bioterrorism attack. The scale of the public response to this attack was not foreseen and threatened to overwhelm the Bioterrorism Response Laboratory’s (BTRL) ability to process and test environmental samples. In a joint effort with the Centers for Disease Control and Prevention and the cooperation of the Department of Defense, a massive effort was launched to maintain and sustain the laboratory response and return test results in a timely fashion. This effort was largely successful. The development and expansion of the facility are described, as are the special needs of a BTRL. The establishment of a Laboratory Bioterrorism Command Center and protocols for sample intake, processing, reporting, security, testing, staffing, and quality assurance and quality control are also described.

Laboratories across the United States have been preparing for the past 5 years for the possibility of civilian populations being the target of bioterrorism (1). The New York City (NYC) Department of Health (DOH) laboratory response plans for bioterrorism changed forever after October 12, 2001, with the knowledge that letters laden with *Bacillus anthracis* spores had been sent through the U. S. Postal Service (2). The original conception of the laboratory’s role in bioterrorism response was not yet fully validated, nor was the need for extensive environmental testing fully appreciated or anticipated. The number of personnel with specialized training was another key factor.

The most probable scenario envisioned a sharp increase in hospital admissions caused by one of the recognized bioterrorism agents (3). By the time the symptoms and bioterrorism agent were diagnosed, the disease was likely to be well established within the local population. Thus, laboratory response would center primarily on human clinical sampling. The scope of required environmental sampling was not fully anticipated and was generally considered to be secondary to the original epidemiologic investigation. Such samples would predominantly consist of samples obtained from the putative source of the exposure.

Although this was the operational scenario, the actual laboratory workload during this event was evenly divided between environmental and clinical samples. However, the amount of labor and materials associated with processing environmental samples for analysis far exceeded that of the clinical samples.

## Background: Laboratory Structure before October 2001

Before October 12, the NYC Public Health Laboratory (PHL) processed one or two suspected bioterrorism environmental samples per month, utilizing a small Biosafety Level 2 (BSL-2) room with two dedicated personnel. In the year before the attack, the PHL received approximately 10 samples, all of which were hoaxes. The laboratory was set up according to Centers for Disease Control and Prevention (CDC) protocols, and staff were trained by CDC on methods for isolating and identifying bioterrorism agents.

The bioterrorism laboratory consisted of a 400–square-foot area designed at BSL-2+ as described in Biosafety in Microbiological and Biomedical Laboratories (4). Entrance to the laboratory was controlled by proximity card access and monitored 24 hours a day by video cameras. In the space were a biosafety cabinet, a fluorescence/phase-contrast microscope, incubators, freezers and refrigerators, a Wallach/Perkin Elmer Victor Time Resolved Fluorescence instrument (The Perkin-Elmer Corp., Norwalk, CN), computers, and necessary laboratory supplies. This configuration provided a comfortable and controlled access space for sample preparation and analysis. Because of the low sample volume, each sample was treated uniquely, and a generalized method for handling numbers of environmental specimens was not considered necessary.

Before October 12, all specimens submitted to NYC BTRL were tested for four priority bioterrorism agents: *B. anthracis* (anthrax), *Francisella tularensis* (tularemia), *Yersinia pestis* (plague), and *Brucella* species (brucellosis). Protocols defined and validated by CDC were used to isolate and identify these agents (5). All specimens tested during that time were culture negative for the four priority bioterrorism agents according to the validated protocols.

## October 12: First Letter Tested Positive

Before *B. anthracis* was identified in letter C from media outlet 1**,** two other letters (A and B) were received and tested by BTRL. Letter A came from media outlet 1, and letter B came from media outlet 2. Letters A and B were tested for the four priority bioterrorism agents and were negative. At the time, the negative result for letter A was somewhat surprising because the patient diagnosed with cutaneous anthrax was employed by media outlet 1. When letter C later arrived at BTRL, it was tested and found to contain a powdery substance that was positively identified as spores of *B. anthracis.* The discrepancy involving the positive results of letters A and C was soon resolved when it was determined that letter C was actually received before letter A at media outlet 1 but was inadvertently placed in a corporate “hate-mail” file and was thus recovered after letter A.

A number of important events took place almost simultaneously after letter C tested positive for *B. anthracis*: a) BTRL was contaminated with *B. anthracis* spores during the sampling process and three BTRL laboratory employees were exposed; b) the news media and U.S. Attorney General John Ashcroft broadcast a message to Americans asking them to report all suspicious mail to their local law enforcement authorities (6); and c) as a result of this increased attention, the sample volume surged and did not abate for another 6 weeks.

These events worked synergistically to complicate NYC DOH’s ability to contend with bioterrorism testing on the scale needed during this crisis. At this time, CDC contacted NYC DOH to offer support and aid. On learning of the situation developing in NYC and the events surrounding the contamination of BTRL, including exposure of employees, the PHL, in conjunction with CDC, instituted several important policies: 1) A Bioterrorism Response Laboratory Command Center was established at PHL to direct and coordinate all bioterrorism laboratory activities and communications; 2) A secure and separate entryway was set up so bioterrorism specimens could enter the PHL building without jeopardizing the safety of PHL building personnel; 3) A separate specimen-receiving area containing a decontamination site was established, and all specimens were doubled bagged and externally decontaminated (sprayed with a bleach solution) before being brought to the testing laboratory for analysis; 4) All environmental bioterrorism specimens were tested by using strict and secure BSL-3 containment and BSL-3 protocols; 5) BTRL personnel exposed in the contaminated laboratory were treated with ciprofloxacin HCl; 6) Extensive infection control and environmental monitoring procedures were set up throughout the PHL building to monitor for *B. anthracis* spores; 7) Security was extensively increased throughout the building’s interior and exterior; 8) During the transition to the new BSL-3 testing facility, samples received for bioterrorism testing were shipped to offsite level C laboratories for analysis; 9) A dedicated database was developed for accepting and tracking bioterrorism specimens and testing results; and 10) CDC and NYC DOH requested a Department of Defense (DOD) Microbiology Response Team to assist with rapid testing of bioterrorism specimens.

After the initial evaluation, the NYC PHL facility was configured to operate 24 hours a day, accepting, processing, and testing samples. Additional laboratory space was identified, consisting of three separate areas for handling and testing bioterrorism samples (two polymerase chain reaction [PCR] units and an enzyme immunoassay [EIA] rapid screening unit). The BTRL coordinator was also appointed to work in conjunction with CDC and DOD teams. Staffs from other units were also redeployed to further assist in the bioterrorism response effort.

## Post–October 12: The Bioterrorism Response Laboratory

Within days of the initial event on October 12, all the essential elements of BTRL were in place. Table 1 describes the transition before and after October 12. Both the types of laboratory activities and their scale changed dramatically. The sample volume increased approximately 3,000 times for both environmental and clinical testing. Not surprisingly, the number of laboratories and ancillary spaces BTRL required increased almost twentyfold, and 25 times more personnel than originally envisioned staffed these additional areas. New instrumentation (i.e., the PCR rapid assays) was brought into BTRL to attempt to process the sample volume more quickly. To supply this dramatic surge, six tons of equipment and supplies was needed. The scale of the operation and the tracking needs threatened to overwhelm the support staff, and a hastily constructed but workable database system was put into place.

**Table 1:** Comparison of the New York City Bioterrorism Response Laboratory requirements before and after October 12, 2001^a,b^

	Before October 12, 2001	After the surge of specimens
Specimen load	1 every 2–3 months	2,700 nasal swabs/2 weeks 3,200 environmental specimens/2 months
Laboratory **s**pace	One room	10 laboratories 3 evidence rooms 4 support areas Command center (suite of offices) Separate storage area for supplies
Staff	2 people rotating on call schedule	>75^c^
Technology	Basic microbiology capabilities • γ phage • DFA	Rapid PCR assays with conventional basic microbiology capabilities
Supplies	General laboratory supplies	6 tons flown to NYC from CDC
Miscellaneous	No database 1 stand-alone computer	Clinical database Environmental database 30 computers linking all areas of the building

^a^When the first letter tested positive for spores of *Bacillus anthracis* was received. ^b^PCR, polymerase chain reaction; DFA, direct fluorescent antibody assay; CDC, Centers for Disease Control and Prevention; NYC, New York City. ^c^From the NYC Public Health Laboratory, CDC, NYC Department of Health, and Department of Defense.

**Figure 1:**
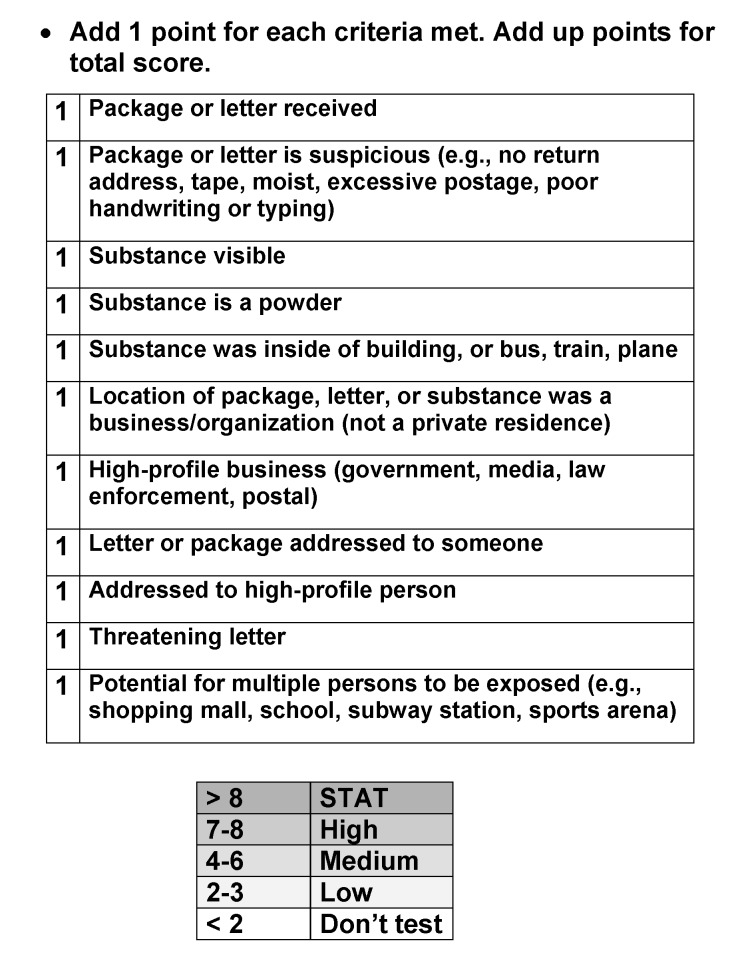
Depiction of the algorithm used to determine the priority of items received for testing at the New York City Bioterrorism Response Laboratory. One of the salient features of the surge was the broad array of items that the laboratory received for testing. Many items contained innocuous powdery substances that are now known to be unrelated to the attack, yet prudent practices required that they be ruled out. The laboratory needed to identify which items were the most urgent and place them first and used this algorithm and other triage methods to prioritize the samples. Samples with 8 out of 11 points or greater were deemed STAT for “highest priority for laboratory testing” and received preferential treatment. Most samples fell into a middle category and were processed in order based on time received.

**Figure 2:**
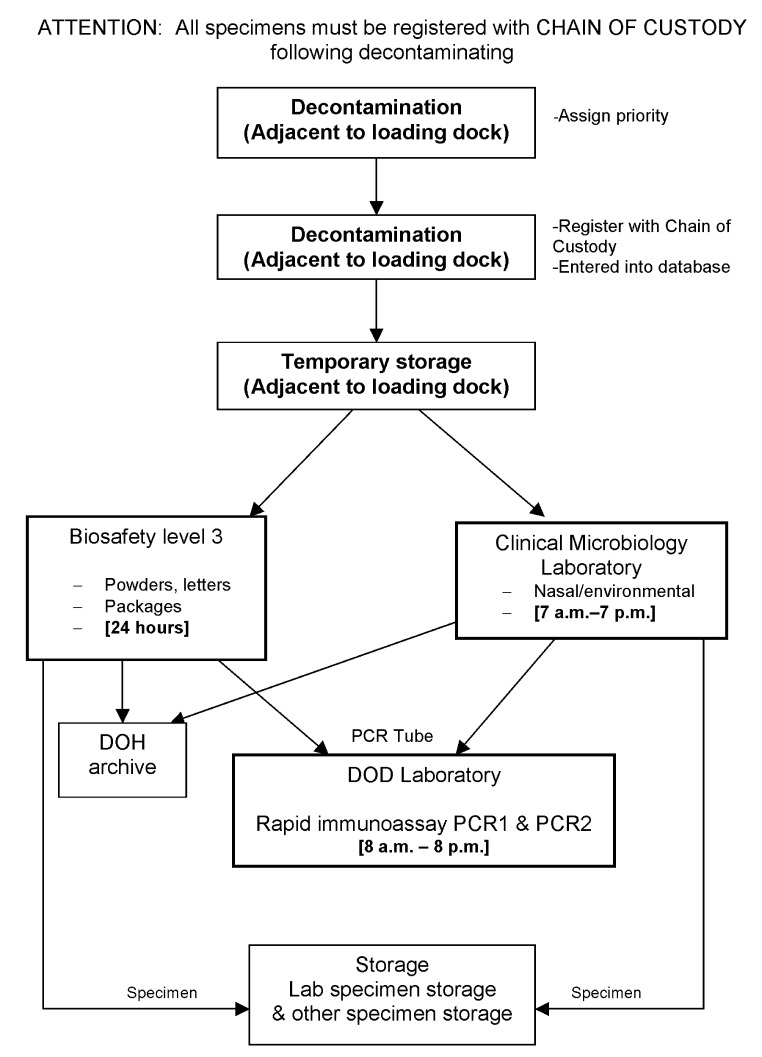
Diagrammatic tracking of an environmental sample through the various units and laboratories as it was processed and tested for anthrax at the New York City Bioterrorism Response Laboratory. The first level of the diagram corresponds to the first floor or the sample intake area. Samples were moved via an elevator to the upper floors of the facility, where they were processed and tested. The final destination of all samples was the storage area. Storage was also a locked and guarded forensic evidence room, and samples released from this area after testing negative for *Bacillus anthracis* were released to the New York Police Department for criminal investigation, return, or disposal.

This sample volume surge was expected to be specimens of human origin (clinical specimens); the need for large-scale environmental sampling and testing had not been anticipated. The clinical laboratories experienced exponential increases in volume but had enough latent capacity to handle the increased workload. Increases in coverage and overtime, plus additional reagents, sufficed to contain the testing volume within manageable limits. Clinical sample processing and tracking were not adversely affected, but environmental sampling was severely hampered. The original testing laboratory was never designed to handle more than perhaps a few samples per day. On the first day of the surge, the laboratory received 34 samples that were considered high priority (Figure 1). Figure 2 shows the flow of a sample as it enters the BTRL. The laboratory can be divided into three main functional entities: 1) a receiving area, which contains a decontamination site in processing area and a secured temporary storage facility; 2) two sampling areas (one each of BSL-2 and BSL-3), containing facilities to unwrap and examine environmental samples and retrieve samples for further analysis (BSL-3), clinical microbiology laboratories, and the PCR laboratories; and 3) locked and guarded storage for samples that had completed the testing protocol and were ready for subsequent distribution as waste, returnable property, or evidence.

## Bioterrorism Response Laboratory: Units, Operation, and Staffing

**Figure 3:**
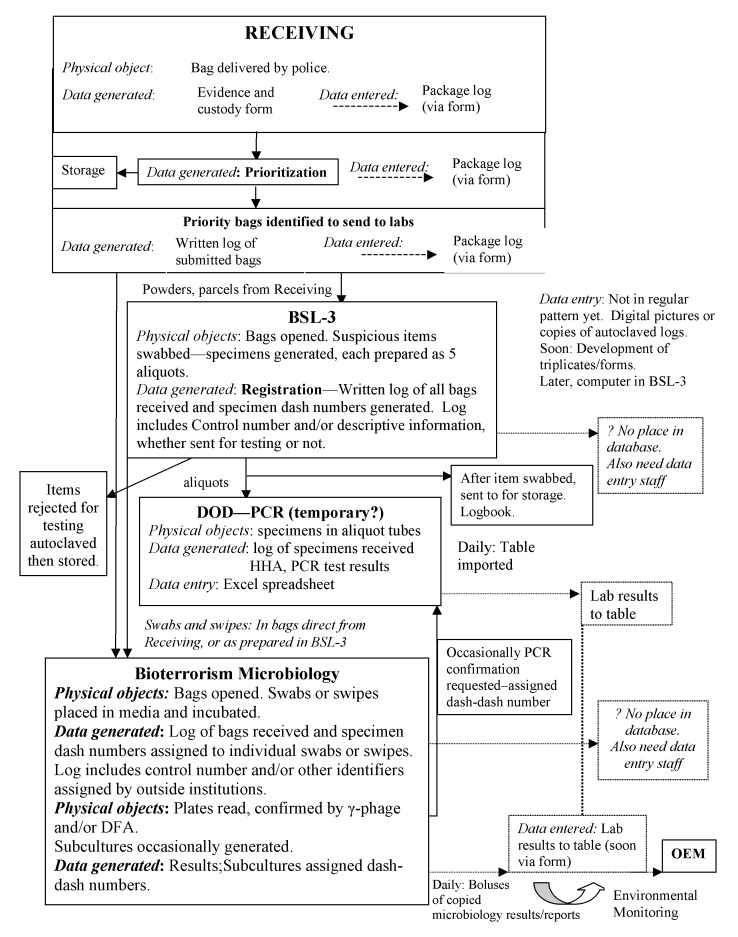
Depiction of the data flow at the New York City Bioterrorism Response Laboratory adopted soon after the surge of isolates after the bioterrorism attack. An access database was developed, and a number of demographic fields and test results were identified and entered. Data retrieved from the Biosafety Level 3 (BSL-3) laboratory after suspicious packages were opened had to be input into the database; the original documentation was modified if any additional information was identified. An attempt was made to monitor all transactions occurring to the sample, which began to make the system unwieldy. The database was modified numerous times and recently was entirely replaced. Most of the comments, such as “no place in database” have been corrected. PCR, polymerase chain reaction; DOD, Department of Defense; HHA, hand-held analysis; DFA, direct fluorescent-antibody assay; OEM, Office of Emergency Management.

Samples were tracked through the system by a specially designed database that reflected the testing status of the sample and its final report status. A large portion of the database was devoted to description and demographics (Figure 3). Table 2 shows a section of a typical spreadsheet.

**Table 2:** A sample section of the data table generated by the tracking system diagramed in Figure 3^a,b^

	Site address^c^	Pick-up date	Intake date	Item description	Testing location	Urgency	Comments	Swab taken?
FBI		10/9/2001	10/9/2001	Envelope (Westchester County)	NYCPHL			No
Hospital A		10/10/2001	10/10/2001	Blood culture	NYCPHL	Stat		No
Hospital B		10/8/2001	10/10/2001	Request for bacterial culture identification	NYCPHL	Stat		No
FBI		10/10/2001	10/10/2001	Petri dish	NYCPHL			No
NYPD			10/11/2001	One express-mail envelope sealed in plastic, addressed to United Nations	NYCPHL	High		No
FBI		10/11/2001	10/11/2001	Plastic bag with white powder; business card.	Wadsworth	Low	not enough info	No
FBI		10/11/2001	10/11/2001	Plastic bag containing one envelope with white powder.	Wadsworth	Low	not enough info	No

^a^From left to right are fields for responder or site of response, site address, date of pick-up, date of intake, bag contents, location of testing, comments, priority, swab taken (yes, no), and patient (if clinical sample). This database allowed the managers to check the progress of sampling and keep track of the “who, what, where, and when” of the samples. ^b^ FBI, Federal Bureau of Investigation; NYCPHL, New York City Public Health Laboratory; NYPD, New York City Police Department; Stat, highest priority for laboratory testing. ^c^Masked for security purposes.

All environmental samples entered the building through the designated bioterrorism intake area. The main function of this area was to provide decontamination, documentation, and security. Samples would be accepted only from designated first responders and law enforcement personnel. Although standard protocols now ensure that the samples brought in for laboratory testing are not externally contaminated with a bioterrorism agent (7), as a prudent preventive measure the outer packaging still needed to be decontaminated in the intake area. A breach in any procedure could compromise the laboratory.

Chain-of-custody documentation was maintained in the intake unit as well as initial entry into the database. All packages came with a test request/manifest document with the data entered and manually maintained at the intake area. Security (provided by NYC DOH Police Department) were present in the area continuously. After passing through decontamination and receiving, packages were held in a nearby temporary storage area until requested by the sampling or testing laboratories.

### Analytical Units

The analytical laboratory was composed of four units: 1) high-containment examination area (BSL-3), where all environmental samples suspected of containing dispersible powders were examined and sampled for further testing; 2) BSL-2 laboratory, for environmental swabs; 3) clinical microbiology, for receiving clinical swabs and analyzing tissue samples; and 4) rapid testing, where the EIA and PCR-based systems were employed, designed to quickly yield preliminary data in advance of the classical microbiology final report.

### Storage

After a sample was tested, it was sequestered in a safe, secure area. Samples testing positive for a bioterrorism agent were stored in a specifically designated, locked storage area separate from the negative samples. All negative samples once reported were handed over to NYPD, where the items were screened for evidentiary purposes. Items not considered evidence were autoclaved and returned to their owners, if valuable. Otherwise, they were discarded. NYPD maintained a log of all transactions and signed off the final disposition on the chain-of-custody form completing the case.

### Laboratory Operation

After a sample passed through the intake area, it either entered the BSL-3 testing area or proceeded as a clinical sample or swab directly into the clinical microbiology unit. Swabs taken from letters, powders, objects, clothes, and other items in the high containment BSL-3 area were plated directly on sheep blood agar (SBA) or transferred onto brain heart infusion broth (BHIB) and incubated there. Another set of samples was taken for rapid testing. These PCR samples were brought out of the containment area and sent to the rapid testing units in separate sample bags decontaminated with a recommended hypochlorite solution (4).

On completion of sampling, the specimen was removed from the biosafety cabinet and taken to the evidence storage area. This procedure posed a problem since it is recommended that items leaving the BSL-3 area be fully decontaminated. Since steam sterilization or chemical decontamination might destroy valuable evidence, we placed the finished item into a sterile biohazard bag that remained uncontaminated on the outside. This newly packaged sample was then removed to the evidence storage area.

### Testing Protocols and Reporting Algorithm<H2>

All testing protocols were adapted from established protocols (8). In short, samples were analyzed by using a rapid screening assay (PCR) to provide preliminary information to health-care providers and law enforcement. However, final disposition of samples was only made after exhaustive identification according to recommended microbiology protocols.

**Figure 4:**
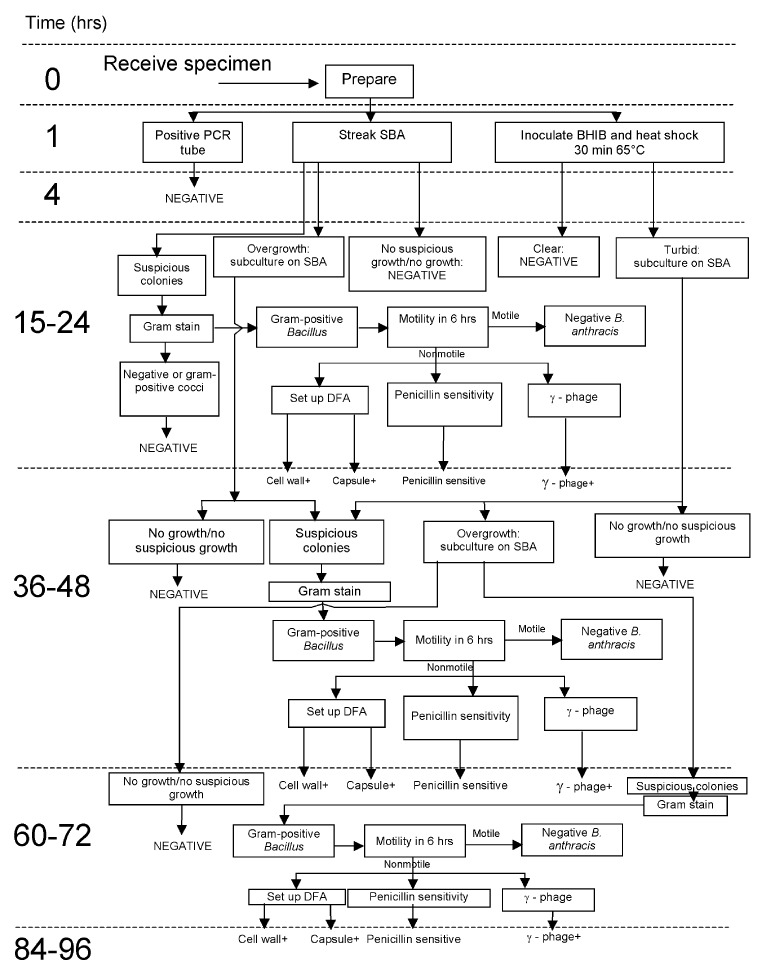
Chart tracking the time needed to report the status of a sample brought in for classical *Bacillus anthracis* testing at the New York Bioterrorism Response Laboratory. Negative samples with no suspicious growth could be reported in 24 hours. However, any samples with growth required some degree of subplating or culturing in brain heart infusion broth (BHIB)**,** were heat shocked, and then tested. Reporting of final results on samples could take 3–4 days. SBA, sheep blood agar; CW, cell wall; CAP, capsule; DFA, direct fluorescent-antibody assay; PCR, polymerase chain reaction; Ph, phage; +, positive.

Figure 4 outlines the workflow through the analytical units. Clinical samples were generally directly plated onto SBA. The environmental samples often were simultaneously transferred into BHIB and heat shocked to kill nonsporulating organisms and enrich for *B. anthracis* spores. A sample was reported as positive only if it had all the following phenotypes: nonmotile; penicillin sensitive; γ-phage positive; and positive by both cell wall and capsule direct fluorescent-antibody assay. Extensive environmental monitoring was performed on the reports before they were released. All negative clinical reports were compiled into a manifest and sent to the Mayor’s Office of Emergency Management, where they were distributed to the appropriate parties.

At the peak of the surge, BTRL was testing 60–100 samples per 24-hour shift. Each sample required, at a minimum, duplicate PCR and an SBA culture. Any growth required the phenotypic testing described above. Most samples were also transferred to BHIB; growth in that medium required phenotypic analysis. The average sample, even if it resulted in a negative finding, required at least 14 separate testing procedures to determine its status.

Processing specimens sampled in the BSL-3 required 30–60 minutes and limited the flow to the microbiology laboratories. Despite any effort to speed testing through the microbiology laboratories, the limiting factor remained the maximal BSL-3 throughput of approximately 40 samples per 24 hours.

### Biosafety and Environmental Monitoring

Normal operations within a clinical microbiology laboratory require routine infection control and quarterly environmental monitoring (9). Because of our experience on October 12 when one laboratory was contaminated, hypervigilance was required to prevent the possibility of further contamination. Our concerns were not only for safety but also for the integrity of the testing process, as stray contamination could seriously mitigate the reliability of the laboratories results.

We instituted a schedule of infection control environmental monitoring. Typical areas that would signal contamination such as door handles, laboratory benches, and hoods, in addition to exposed skin of technical personnel, were routinely sampled each day. Approximately 70 data points were routinely sampled from the various bioterrorism units around the facility, including the intake area, elevators to the BSL-3, the BSL-3 (all three shifts), microbiology laboratories, PCR laboratories, and all personnel associated with operations. Additional areas sampled frequently were the storage room and various corridors in the facility.

### Laboratory Staffing

With minor variations, the PHL had a fully functional BTRL running 24 hours a day, 7 days a week within the first week. Approximately 75 personnel from DOH, CDC, DOD, and other organizations worked each shift. Scheduling was further complicated because DOH personnel had to be borrowed from other testing units and could not be dedicated to the bioterrorism effort alone.

Staffing during the surge consisted of DOH personnel along with the CDC emergency team. The DOD Microbiology Rapid Response Team that supported testing in the microbiology and BSL-3 sampling unit filled a number of slots. The DOD was also completely responsible for the rapid testing units.

A unique aspect of the staffing requirements for BTRL was the need for extensive security. The DOH Police Department carried out this function. Officers were present in the intake area and guarded the sensitive testing and storage areas around the clock. They were responsible for maintaining the chain of custody and for initial intake of information after the first response units brought samples to the laboratory. In addition, officers increased all aspects of security for the building with extensive identification (ID) checks, closed circuit television surveillance, and maintenance of ID cards. Essentially, the DOH Police Department continuously monitored all personnel movement in the facility.

### Physical Security

 Physical security concerns became paramount because samples brought to the BTRL were also potential criminal evidence and therefore required special precautions (e.g., chain of custody, locked or guarded storage areas) to protect their integrity. To accomplish these security goals, the DOH Police Department augmented laboratory security by increased background investigation of personnel, extensive implementation of physical security procedures, and oversight of laboratory accessions and evidence containment. The DOH Police Department investigated unusual work practices, breaches of confidentiality, and safety issues with an eye to possible lapses in security.

 Physical security was enhanced by the use of ID cards, restricted area badges, and a sign-in logbook. Only one entrance was open to the public, while another entrance was designated for bioterrorism sample accession. Card access was instituted for all sensitive areas such as the testing laboratories and the evidence room. This system allowed for tracking of users and limiting such use to specific personnel at specific times. All card and badge access was tracked. All entrances, elevators, emergency exits, and sensitive laboratories were monitored continuously by closed-circuit television, and all transactions were recorded.

 The use of biological, chemical, or radiologic materials with the intent of causing injury or death is a crime, and the instrument used and swabs or specimens obtained from the crime scene are potentially evidence (10). The DOH Police Department maintained responsibility for accepting and storing proper evidence to maintain its integrity as it was transferred from law enforcement into the laboratory for testing. Custody containment, which ensured the integrity of the evidence for prosecution, was also maintained by the DOH Police Department.

## Conclusion

The events of September 11, 2001, placed New York City on high alert immediately (11). On the heels of this tragedy, the City became the target of a bioterrorism attack (12). NYC DOH, as part of the city’s emergency response network, was extensively involved with the mitigation of both these catastrophes. The laboratory had recent experience in public health emergencies such as the West Nile virus outbreak (13) and the 1999 bottled-water scare.

 Although PHL had chain-of-custody experience through its Toxicology and Environmental Laboratories and outbreak testing during the West Nile outbreak, nothing could have prepared the laboratory for the events of October 2001. Nevertheless, staff outfitted the laboratory within days to accept, test, report, store, and return evidence from thousands of environmental and clinical samples.

 In the months after the crisis, BTRL still receives about five suspicious samples per week. Samples are now routinely tested for the four priority agents, and plans have been finalized for dedicated laboratory space designed by using the lessons learned from October 2001.

 Nevertheless, before October 2001, we thought we were prepared to confront an event on the scale of this bioterrorism attack. An important lesson from this experience is that, despite all additional precautions and enhancements made to the laboratory and the response network, another attack, if and when it occurs, will present further surprises. While the laboratory has now institutionalized weapons of mass destruction testing to be performed as part of routine surveillance (e.g., testing of drinking water), potential means and targets for future attacks cannot be perfectly forecast. Vigilance and continued emphasis on flexibility, creativity, and the ability to rapidly expand our response, as needed, to bioterrorism events and the surprises they present will determine our effectiveness and ultimate success.
